# 28. The Paradox of an Integrated MAT OPAT Program

**DOI:** 10.1093/ofid/ofab466.230

**Published:** 2021-12-04

**Authors:** Paula Eckardt, Jessica Reed, Claudia P Vicencio, Alberto Augsten

**Affiliations:** 1 MHS, weston, FL; 2 Memorial Healthcare System, Hollywood, Florida

## Abstract

**Background:**

Patients with substance use disorders (SUD), specifically opioid use disorder (OUD) and injection drug use (IDU) utilize healthcare resources for prolonged inpatient treatment of serious infections stemming from their addictions. For a variety of reasons, physicians treating these patients refuse to send these patients home to receive outpatient parenteral antimicrobial therapy (OPAT), and instead keep the patient in the hospital for several weeks or longer to complete treatment for the injection-related infections. Patients who do not have history of IDU are sent home with a PICC line to receive OPAT once they are no longer acutely ill and therefore no longer meet criteria to remain inpatient, which is the established standard of care. Patients with OUD and IDU are not allowed the same standard of care, and furthermore do not receive adequate, if any, therapy for their primary problem and reason for their serious infection – the addiction.

Flow chart of the MAT-OPAT process

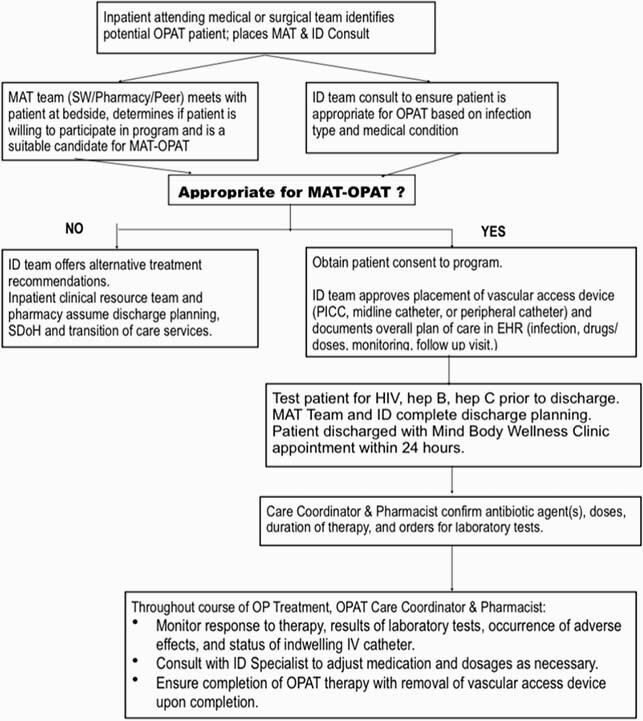

**Methods:**

Medication-assisted treatment (MAT) with buprenorphine-naloxone has been approved for treating adults with opioid use disorder as part of a comprehensive treatment program that also includes counseling and behavioral therapy. Until now in our healthcare system there has been no comprehensive and integrated program to safely discharge patients with OUD and IDU to receive OPAT via a PICC line, while simultaneously treating their addiction. We describe the implementation of a MAT-OPAT program. Please refer to the chart included.

**Results:**

We present a successful case of a 36-year-old male with a history of endocarditis associated with IV drug use and the intervention of the Healthcare System to link the patient to appropriate Infectious disease, behavioral health and medication adherence treatment for opioid abuse. The patient completed the IV antibiotic therapy and remained enrolled in the behavioral health program with a successful outcome.

**Conclusion:**

MAT-OPAT implementation in large healthcare system with continuous outpatient support that includes Infectious Disease services, behavioral health and drug abuse rehabilitation therapy can be a successful strategy to minimize readmisión, cost and complications in patients with history of IV drug use and infections that require prolonged intravenous antibiotic therapy.

**Disclosures:**

**All Authors**: No reported disclosures

